# The implications of a pathological complete response of the primary tumour after neoadjuvant chemotherapy for breast cancer on axillary surgery

**DOI:** 10.1186/s43046-021-00061-9

**Published:** 2021-02-08

**Authors:** Mina M. G. Youssef, Ahmed A. Metwally, Tamer M. Manie

**Affiliations:** 1 Department of Breast Surgery, Surgical Oncology, National Cancer Institute—Cairo University, Giza, Egypt; 2 Department of Breast Surgery, Norfolk and Norwich University Hospital, Colney Lane, Norwich, NR4 7UY UK

**Keywords:** Neoadjuvant chemotherapy, Complete pathological response, Management of axilla

## Abstract

**Background:**

Management of the node-positive axilla after neoadjuvant chemotherapy is controversial. The aim of this study is to predict the group of patients who may require a less invasive approach for axillary management. One possible group are patients with pathological complete response of the primary after chemotherapy.

**Results:**

A unicentral retrospective cohort study including all breast cancer patients with axillary node metastases at presentation who received neoadjuvant chemotherapy resulting in pathological complete response. Pathological complete response in the axillary lymph nodes was recorded. A correlation between the response in the primary tumour and the lymph nodes was assessed. A subgroup analysis was conducted for different biological groups.

Complete response was seen in the axillary nodes in 80.5% of patients. Patients with lobular cancer were less likely to show a similar response in the axilla as the primary tumour (*p* = 0.077). A higher incidence of axillary response was observed in HER2-positive tumours (*p* = 0.082). All patients with grade 3 tumours achieved complete response in the axilla (*p* = 0.094). Patients with negative or weak positive hormone receptor status had a significantly higher rate of complete response in the axilla compared to strongly positive hormone receptor status (OR, 7.8; 95% CI, 1.7–34.5; *p* = 0.007).

**Conclusion:**

A less invasive axillary surgery may be safely recommended in selected group of node-positive patients after neoadjuvant chemotherapy when the primary tumour shows complete response. This group may include HER2-positive, ER-negative and grade 3 tumours. Less response is expected in ER-positive and lobular carcinoma even with complete response in the primary.

## Background

Neoadjuvant chemotherapy (NACT) is a well-established treatment modality in breast cancer [1].

Its role has expanded to include early-stage breast cancer in addition to the standard indications for inoperable and locally advanced tumours [2].

Potential benefits include downstaging the primary tumour to allow for more conservative surgical management [3], testing and monitoring the response of new drugs in vivo [4] and allowing additional time for genetic tests (e.g., BRCA1/2) whose results can affect the surgical management [5].

Although previous randomized controlled studies showed no difference in survival between neoadjuvant and adjuvant chemotherapy [6, 7], it is now understood that achieving a pathological complete response (pCR) is a surrogate for a favourable prognosis and a better disease-free and survival outcome [8]. This pCR is mostly seen in HER2-positive and triple-negative subtypes [9].

Management of the axilla following NACT has always been a topic for debate [10, 11]. Management of a clinically node-negative (N0) axilla with a sentinel node biopsy at the time of surgical resection is widely acceptable as a standard of care [12].

However, the management of a clinically node-positive (N1+) axilla is more controversial [13]. Many studies have tried to address this issue [14–16]. The ABS guidelines for management of the axilla after neoadjuvant chemotherapy cautiously recommend a targeted axillary dissection (TAD) approach [17] in selected clinical situations.

Some authors tried to establish a relation between the response of the primary tumour and the axillary lymph nodes in initially node-positive patients [18, 19]. The aim is to predict the group of patients who may require a less invasive approach for axillary management after NACT [18]. One possible group is the patients who develop complete pathological response (pCR) in the primary tumour after NACT [18].

This is a retrospective cohort study of axillary lymph node-positive breast cancer patients at presentation who showed a pCR of the primary tumour after NACT.

The aim of the study was to establish a relationship between the pCR in the primary tumour and the response in the axillary lymph nodes. We looked at the pathological changes in the axillary lymph nodes status within the group of patients with pCR in the primary.

The study questioned whether a pCR in the primary tumour implied a corresponding downstaging of the axillary lymph nodes status.

Establishing this relationship aims to safely identify a potential group of patients with axillary lymph node metastases at presentation where the axillary surgical staging procedure required may be less invasive after completion of NACT.

## Methods

A retrospective cohort study including all breast cancer patients with axillary lymph node metastases at presentation who received neoadjuvant chemotherapy (NACT) resulting in pathological complete response (pCR) between January 2013 and December 2017 at a high-volume tertiary referral centre operating on 2000 breast cancer patients per year. Approval of the Institutional Review Board was obtained for the study.

Patients who presented with breast cancer involving the axillary lymph nodes (N1+) and received NACT leading to a pCR following that were included. Breast cancer patients with negative axillary lymph nodes (N0) were excluded. Other exclusion criteria were patients with distant metastatic disease (M1+) at presentation and patients with N1+ disease, who received NACT but showed any response less than pCR following that.

All patients were diagnosed with the standard triple assessment, with additional imaging (e.g., MRI) done whenever required. Hormone receptor (HR) and HER2 status were identified in the core biopsy through immunohistochemistry (IHC). HR receptors quick score was calculated. HER2 was assessed using FISH when the IHC was equivocal.

Based on the tumour biology features, patients were classified into 4 groups: Luminal A (HR+, HER2−), luminal B (HR+, HER2+), HER2 enriched (HR−, HER2+) and triple negative (HR−, HER2−).

Axillary lymph node involvement at presentation was established by ultrasound scan of the axilla with a cortical thickness more than 3 mm and loss of the fatty hilum. At the time of this study, no biopsy was performed.

Each case was discussed at the multidisciplinary team (MDT) meeting.

NACT regimen consisted mainly of a combination of anthracyclines and taxanes or FEC. All HER2-positive patients received NACT and trastuzumab with the latter continued after surgery.

After completion of NACT, patients underwent breast imaging to assess the response and to guide the surgical planning. Surgical options included mastectomy with or without immediate reconstruction, and breast conserving surgery (BCS). All patients underwent axillary node clearance.

Pathological complete response (pCR) of the primary tumour was established as absence of invasive or in situ cancer (Miller and Payne score of 5) [20]. pCR in the axilla was diagnosed when no residual tumour was found in the lymph nodes, including isolated tumour cells and micrometastases.

pCR in the axillary lymph nodes was recorded and correlated with the initial status at presentation. A correlation between the response in the primary tumour and the lymph nodes was assessed. A subgroup analysis was conducted for different biological groups.

Statistical analysis was done using IBM SPSS® Statistics version 22 (IBM® Corp., Armonk, NY, USA). Numerical data were expressed as mean and standard deviation or median and range as appropriate. Qualitative data were expressed as frequency and percentage. Pearson’s Chi-square test or Fisher’s exact test was used to examine the relation between qualitative variables. For not normally distributed quantitative data, comparison between the two groups (N0 and N+) was done using Mann-Whitney test (non-parametric *t* test). Multivariate analysis was done using logistic regression forward method for the significant factors affecting lymph node positivity postoperatively on univariate analysis. Odds ratio (OR) with it 95% confidence interval (CI) were used for risk estimation. All tests were two-tailed. A *p* value < 0.05 was considered significant.

## Results

Three hundred sixty-seven (367) patients received NACT at the study centre between January 2013 and December 2017. Of those patients, 77 (20.9%) achieved pCR in the breast and were included in the study.

pCR was seen in the axillary lymph nodes in 62 (80.5%) of this cohort of patients. In the remaining 19.5% (15 patients), pCR was not observed in the axilla.

Clinicopathological characteristics of the study cohort are listed in Table 1.

**Table 1:** Clinicopathological characteristics

	Total group	N0	N1	*p* value
Number	77	62	15	
Mean age	47.4 (22–75)	47.3 (22–75)	47 (34–71)	0.918
Tumour stage				
**Stage IIB**				
T2 N1	15	13	2	
**Stage IIIA**				
T2 N2	1	0	1	
T3 N1	25	18	7	
T3 N2	12	11	1	
Total	38	29	9	
**Stage IIIB**				
T4a N1	13	11	2	
T4a N2	9	8	1	
Total 22		19	3	
**Stage IIIC**				
T1 N3	1	0	1	
T3 N3	1	1	0	
Total	2	1	1	
Histological type				
IDC	68	57	11	0.077
ILC	6	3	3	
Others	3	2	1	
Molecular subtype				
Luminal A	18	12	6	0.232
Luminal B	12	10	2	
Her2+	24	22	2	
TNBC	23	18	5	
Mean tumour size	4.1 cm (1.2–11)	4.2 cm (2.5–11)	3.8 cm (1.2–7)	0.876
Mean number of LNs	12.8	12.9	12.4	0.806
Histological grade				
Grade 2	57	45	12	0.094
Grade 3	11	11	0	
Unknown	9	6	3	
Surgery				
MRM	64	54	10	0.058
CBS	13	8	5	

The median age of patients was 47 years (range 22 to 75 years). The median tumour size before neoadjuvant chemotherapy was 4 cm (range 1.2 to 11 cm). The distribution of the nodal stage at presentation (before NACT) ranged between N1 (53 patients, 68.8%), N2 (22 patients, 28.5%) and N3 (2 patients, 2.5%).

The most common clinical stage was IIIA (43%). Ninety percent of the patients showed an invasive duct carcinoma and 10% had a lobular cancer. The molecular subtypes were HER2 enriched (31%), TNBC (29.8%), Luminal A (23.3%) and Luminal B (15.5%).

NACT regimen consisted mainly of a combination of anthracyclines and taxol (48%) and FEC regimen (42%). Other less commonly used regimens included FAC (3%), taxane + carboplatin (2%), gemzar + carboplatin (1%) and taxanes + endoxan (1%)

Sixty-four patients underwent mastectomy (83%), with 5 of them having immediate reconstruction. All the patients in the study had axillary clearance, with a mean number of 12 lymph nodes harvested.

Sixty-two patients (80.5%) had a pCR in the axilla demonstrated in the final histological assessment. Age, pre-chemotherapy size of the primary tumour and number of lymph nodes harvested in the axillary clearance had no effect on the development of pCR in the axilla (*p* = 0.918, *p* = 0.876, *p* = 0.806).

Patients with lobular cancer were less likely to show a similar pCR in the axillary lymph nodes as the primary tumour than those with invasive ductal carcinoma (*p* = 0.077).

On the contrary, a higher incidence of axillary lymph nodes pCR was observed in HER2-positive tumours compared to other biological types including HER2-enriched and luminal B subtypes (*p* = 0.082).

All patients with grade 3 tumours achieved pCR in the axilla, compared to 78.9% of those with grade 2 tumours (*p* = 0.094).

For the statistical purpose of determining the effect of hormone receptor positivity, we grouped patients with negative and weak positive hormone receptors together, compared to patients with intermediate and strong hormone receptor positivity.

On multivariate logistic regression analysis, the independent factor that significantly affected the lymph node positivity postoperatively was the degree of hormone receptor positivity. Patients with negative or weak positive hormone receptor status had a significantly higher rate of pCR in the axillary lymph nodes compared to those with intermediate or strong positive hormone receptor/status (OR, 7.8; 95% CI, 1.7–34.5; *p* = 0.007).

## Discussion

This study addresses the question of whether a complete pathological response observed in the primary tumour after NACT can predict a similar response in the axillary lymph nodes and therefore allow for less invasive surgical approach for axillary staging.

The rate of pCR of the breast in our study was 21%, which is in keeping with the 22% from the meta-analysis by Cortazar and Geyer [8] and the 24% of the Dutch multicentre trial [19].

Patients who achieved pCR in both the breast and axilla comprised 16.9% of all patient population, which is similar to results from Schipper et al.’s 17.5% [21] and Fayanju et al.’s 19% [22] studies that included 20,000 patients.

In our study, pCR of the primary tumour was associated with change in the stage of the axillary lymph nodes after NACT. Patients who had presented initially with positive axillary lymph nodes and showed a pCR in the primary tumour mostly showed similar response pattern in the axilla after clearance.

Morgan et al. [23] showed similar results in their study which included 83 patients with pathological axillary lymph nodes who received NACT. Twenty-one patients had pCR in the breast. Of those, 17 (80%) had also a complete response in the axilla which is the same percentage as our study.

Another study by Tadros et al. [24] included the same number of patients as our study yet had a higher rate of pCR in both the breast and axilla (89.6%). This difference can be attributed to the fact that the study only included HER2-positive and triple-negative subtypes. The multicentre study by Samiei et al. [19] reports a 45% of patients (245/544) achieving pCR in both breast and axilla.

The pCR in the primary corresponded to a similar response in the axillary lymph nodes in most of the study cohort.

There seem to be a variation among different groups of patients treated with NACT in terms of the expected response in the axilla following pCR of the primary.

The presence of pCR in both the breast and axilla depended largely on the molecular subtype of the tumour. The commonest subtype was HER2 enriched (91.7%) followed by Luminal B (83.3%), triple-negative breast cancer (78.2%) and finally Luminal A (66.7%). These results are in concordance to large database study by Barron et al. [18] that included 30,821 patients who received NACT. Among the 3128 patients who achieved pCR in both the breast and axilla, the most common molecular subtypes were HER2 enriched (88.7%), Luminal B (86.7%), TNBC (85.9%) and Luminal A (69.5%).

A similar good response was observed in grade 3 tumours as well, which was also witnessed in the multicentre study by Samiei et al. (*p* = 0.045) [19].

The response was inversely proportional to the degree of HR positivity, with HR-positive tumours less likely to show pCR in the axillary lymph nodes following complete response in the primary. This same observation which was described in the meta-analysis by Houssami et al. [25] and the pooled analysis by Cortazar et al. [26].

Patients with lobular cancer were less likely to show similar pattern.

There have been previous published literature aiming at predicting patients with axillary pathological complete response following NACT.

Schipper et al. [21] proposed a model that included patient age, clinical tumour stage, histological subtype, hormone receptor and HER2 status, as well as receiving trastuzumab and taxanes. Their model had a specificity of 43% and PPV of 65%.

Kim et al. [27] proposed a prognostic nomogram to predict who achieve axillary pCR following NACT. The nomogram items included patient age, initial clinical T stage, clinical nodal stage, tumour grade, ER, HER2, Ki67 expression, clinical tumour and nodal response and regimen of NACT.

The factors included in that nomogram seem to correlate to the factors that we have identified as influencing the pCR in both breast and axilla.

To the best of our knowledge, this is one of the few studies looking specifically at the group of patients who showed pCR in the primary tumour to establish correlation with the axillary response. This group is identified as a potential group where axillary staging could be less invasive. It is possible that this group of patients can be offered a less invasive axillary staging procedure such as sentinel node biopsy or target axillary dissection.

We recognize many limitations to our study. A main limitation is the fact that the axillary lymph node positivity was established pre-chemotherapy with ultrasound scan and without a biopsy. Although the signs of a malignant lymph node on ultrasound are highly reliable [28], most centres depend on a core biopsy to establish this diagnosis. At the time of studying this cohort at our centre, the radiological diagnosis was considered adequate to establish nodal involvement. It is now clear that most centres require core biopsy to confirm axillary nodes involvement.

Another main limitation is the lack of availability of target axillary dissection approach (TAD) [29]. In our centre, this approach has not been adopted yet. Selected patients with low disease burden in the axillary lymph nodes initially could have been offered this approach in an attempt to spare them axillary clearance. However, most of the patients included in this study had presented with heavy nodal involvement initially (at least 3 pathological lymph nodes) and therefore would not have been suitable for TAD according to the current guidelines [17] even if it were available.

Another limitation is the retrospective nature of the study. This is compensated by the high-quality data accurately obtained from pathological reports to minimize a recall bias. The small number of patients included in the study reflects the real-life percentage of patients with initial nodal metastases who develop pCR (13–23% [19, 22]) in the primary.

Based on the findings in our study, a less invasive axillary staging approach may be safely recommended in a selected group of node-positive patients after NACT when the primary tumour shows pCR. This group may include HER2-positive, HR-negative and grade 3 tumours. Less response is expected to be observed in the axillary lymph nodes in HR-positive and lobular carcinoma even with pCR in the primary tumour.

However, more body of evidence to support this hypothesis is needed. A target axillary dissection (TAD) approach may be considered in early small nodal disease [29] but should be discussed carefully within the MDT.

Further studies are needed to attempt at selecting groups of node-positive patients who can be safely offered less invasive axillary staging surgery after NACT. A selected group of patients among those who show pCR in the primary tumour are one of the possible groups.

## Conclusion

A correlation between a pathological complete response in the primary tumour and the axillary lymph nodes is more likely to be seen in patients with grade 3, HER2-positive or HR-negative tumours. In this group, a more conservative approach to axillary surgery can be safely adopted, sparing them a full axillary clearance.

This group can be identified at MDTs, therefore avoiding axillary clearance in patients who show pathological complete response after neoadjuvant chemotherapy who were initially node positive.

## Data Availability

The raw data and materials are available upon request.

## Abbreviations

BCS: Breast conserving surgery
CI: Confidence interval
HR: Hormone receptor
IHC: Immunohistochemistry
MDT: Multidisciplinary team
N0: Clinically node negative
N1+: Clinically node positive
NACT: Neoadjuvant chemotherapy
OR: Odds ratio
pCR: Pathological complete response
TAD: Targeted axillary dissection
